# Childhood-Onset Systemic Lupus Erythematosus Research over the Past Decade in Japan

**DOI:** 10.3390/children13020250

**Published:** 2026-02-11

**Authors:** Tasuku Tamai, Hiroyuki Wakiguchi, Kenji Ihara

**Affiliations:** 1 Department of Pediatrics, Oita University Faculty of Medicine, 1-1 Idaigaoka Hasama-machi, Yufu 879-5593, Oita, Japan; m08065tt@oita-u.ac.jp (T.T.); k-ihara@oita-u.ac.jp (K.I.); 2 Division of General Pediatrics and Emergency Medicine, Department of Pediatrics, Oita University Faculty of Medicine, 1-1 Idaigaoka Hasama-machi, Yufu 879-5593, Oita, Japan

**Keywords:** belimumab, glucocorticoid-sparing therapy, lupus nephritis, macrophage activation syndrome, mycophenolate mofetil, type I interferon

## Abstract

**Highlights:**

**What are the main findings?**

- Research on childhood-onset SLE in Japan has grown notably since 2015.
- Lupus nephritis and type I interferon signaling remain central research themes.

**What are the implications of the main findings?**

- Accumulating evidence supports GC-sparing approaches in childhood-onset SLE.
- Multicenter collaboration is essential to strengthen pediatric-specific evidence.

**Abstract:**

**Background**: Childhood-onset systemic lupus erythematosus (cSLE) is a rare, serious autoimmune disease characterized by multiorgan involvement and long-term morbidity. Although several studies have examined this condition in Japan, a comprehensive summary of recent findings remains lacking. **Methods**: PubMed was searched for Japanese publications on cSLE published between 2015 and 2025, including clinical studies, case reports, translational research, basic science studies, systematic reviews, clinical practice guidance, and transition care guidance. **Results**: Sixty publications met the inclusion criteria: 20 clinical studies, 30 case reports, 6 translational studies, 1 basic science study, 1 systematic review, 1 clinical practice guidance, and 1 transition care guidance. Most clinical studies were retrospective, although multicenter and registry-based designs have increased in recent years. Lupus nephritis remained the primary research focus, with accumulating evidence supporting mycophenolate mofetil, tacrolimus, and early belimumab as glucocorticoid (GC)-sparing approaches. Case reports illustrated the broad clinical spectrum of cSLE, with hematological and vascular complications being the most frequently reported. Translational studies highlighted the pathogenic role of type I interferon signaling and cytokine dysregulation, particularly in macrophage activation syndrome. Despite these advances, prospective studies and standardized assessment methods for pediatric-specific practice remain limited. **Conclusions**: Over the past decade, cSLE research in Japan has contributed to a deeper understanding of its clinical and immunological characteristics. However, treatment-related complications and long-term organ damage remain important challenges. Continued multicenter collaboration and domestic data accumulation may strengthen the evidence base, facilitate optimization of GC-sparing approaches, improve clinical management, and provide background information to support future discussions on clinical practice guidelines.

## 1. Introduction

Childhood-onset systemic lupus erythematosus (cSLE) is a more acute and severe form of autoimmune disease than adult-onset SLE and often leads to long-term organ damage [1,2]. The global prevalence of SLE ranges from 13 to 7713.5 per 100,000 individuals [3]. The epidemiology of SLE shows marked geographic and ethnic variation globally, with prevalence and incidence being higher among individuals of African, Asian, and Hispanic ancestry [1,4]. In Japan, the prevalence of SLE in the total population has been reported to be approximately 60 per 100,000 individuals [5]. Data from Asian countries indicate a higher risk of SLE-related complications, particularly severe renal involvement, underscoring the clinical importance of this disease [1,4]. In 2018, the Pediatric Rheumatology Association of Japan and the Pediatric Rheumatology Subcommittee of the Japan College of Rheumatology developed and published clinical practice guidance for cSLE in Japanese, aiming to optimize diagnosis, assessment, and treatment based on disease severity and the risk of organ involvement [2,6]. This guidance has helped specialists align their treatment goals, provided common recommendations to standardize clinical practice, and improved the quality and extent of collaboration among specialists. The Japanese diagnostic guidance for cSLE (JDG) included in the clinical practice guidance was developed to reflect the distinct clinical characteristics of SLE in Japanese children. Children with SLE in Japan often exhibit lower serum complement levels than those of adults. Accordingly, the JDG define cSLE as meeting four or more of 12 items, based on the 1997 American College of Rheumatology classification criteria (ACR) [7], with “hypocomplementemia” added as the twelfth item. In 2015, hydroxychloroquine (HCQ) and, in 2016, mycophenolate mofetil (MMF) were approved for the treatment of SLE and lupus nephritis (LN), including childhood-onset cases, in Japan, facilitating the dissemination of internationally accepted standard therapies. Since 2019, several biologics have become available for children with SLE and LN. Despite these advances, clinical practice guidance for cSLE in Japanese has not been updated since its publication in 2018 [6]. While the shared use of standardized guidance has facilitated more consistent clinical practice and strengthened collaboration among specialists, advances in diagnostic tools, the introduction of biologics, and changes in clinical practice and healthcare settings underscore the need for updated guidance. Considering that the spread of internationally recognized standard treatments has been promoted since 2015, understanding the types of clinical research and case reports accumulated domestically over the past decade is essential to inform future revisions. This review systematically identified and analyzed cSLE studies published in Japan between 2015 and 2025. The publications were categorized as clinical studies, case reports, translational studies, basic science studies, clinical practice guidance, and transition care guidance. By summarizing published reports from the past decade, this review aimed to describe research trends and unresolved issues, and identify areas that may help inform future discussions on clinical practice guidelines in Japan.

## 2. Materials and Methods

### 2.1. Literature Search

A literature search was conducted using the PubMed database. Appropriate search terms combining Medical Subject Headings (MeSH) and free-text keywords were used to identify studies on SLE and LN in pediatric populations in Japan. The main MeSH terms included “Systemic Lupus Erythematosus,” “Lupus Nephritis,” “Child,” and “Adolescent,” which were combined with free-text terms such as “pediatric,” “Japan,” and “Japanese.”

An example of the PubMed search strategy was as follows: (“Systemic Lupus Erythematosus” [MeSH] OR “Lupus Nephritis” [MeSH]) AND (“Child” [MeSH] OR “Adolescent” [MeSH] OR pediatric) AND (Japan OR Japanese). The search was limited to articles published between 1 January 2015, and 15 September 2025 (last accessed on 16 September 2025).

After duplicate records were removed, titles and abstracts were screened to identify publications related to cSLE. In addition to the database search, hand searching of the reference lists of relevant articles and reviews was performed to identify additional publications.

### 2.2. Inclusion and Exclusion Criteria

The inclusion criteria were: (1) studies involving patients diagnosed with SLE at ≤18 years of age. Studies that included adult patients were eligible only if the pediatric subgroup was clearly specified. Eligible publications comprised clinical studies, case reports, translational studies, basic science studies, clinical practice guidance, and transition care guidance issued by medical institutions or research groups based in Japan.

The exclusion criteria were studies that (1) focused on adult-onset SLE in Japanese patients; (2) were multinational or international collaborative studies in which data specific to Japanese pediatric patients could not be independently extracted from the published reports; (3) examined SLE cohorts outside Japan; (4) were reviews, conference proceedings, or consensus statements; or (5) did not focus on patients with SLE.

### 2.3. Data Extraction and Classification

Data were collected using a standardized data sheet that had been prepared before the review. For case reports, the authors, publication year, major complications, and clinical course were recorded. The histological classification of LN followed the International Society of Nephrology/Renal Pathology Society (ISN/RPS) system [8,9]. For clinical studies, information on the authors, publication year, study design, study setting (single-center, multicenter, or national database), sample size, study objectives, and main findings were extracted. For translational studies, basic science studies, systematic reviews, clinical practice guidance, and transition care guidance, the authors, publication year, study objectives, and main findings were summarized. All data were independently extracted by two reviewers, and any discrepancies were resolved through discussion until consensus was reached.

## 3. Results

**Figure 1:**
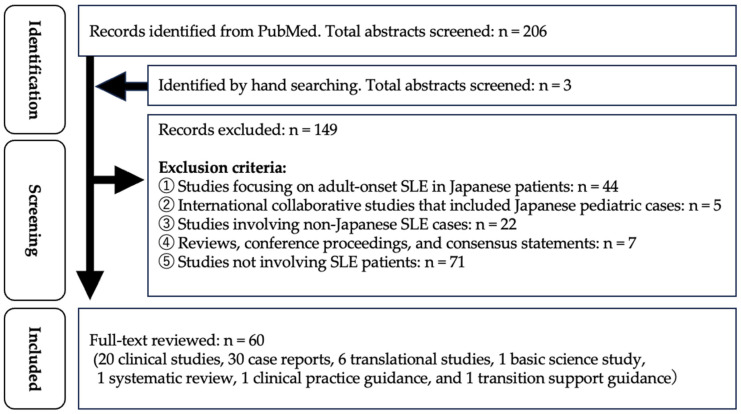
Literature search flow diagram. SLE, Systemic lupus erythematosus.

Over the past decade, 20 clinical studies of cSLE have been published in Japan. Furthermore, 30 case reports, 6 translational studies, 1 basic science study, 1 systematic review, 1 clinical practice guidance, and 1 transition care guidance were identified and included in our review (Figure 1).

### 3.1. Clinical Studies

All 20 publications on cSLE in Japan between 2015 and 2025 were retrospective clinical studies (Table 1) [5,10,11,12,13,14,15,16,17,18,19,20,21,22,23,24,25,26,27,28]; no prospective or interventional studies were identified. Eight were single-center studies, 10 were multicenter (including 2 registry-based), and 2 were national database analyses. Two registry-based studies utilized national databases: one used the Japan Renal Biopsy Registry, maintained by the Japanese Society of Nephrology, which collects nationwide renal biopsy data from adults and children; the other used the Pediatric Rheumatology International Collaboration Unit Registry (PRICURE v2), operated by the Pediatric Rheumatology Association of Japan, a national registry for pediatric rheumatic diseases [14,17]. Earlier studies (2015–2017) were mostly single-center and retrospective; however, since 2019, there has been a gradual shift toward multicenter and database-based research. The growing number of collaborative studies between 2023 and 2025 reflects an expanding national research network. Research themes were dominated by treatment-related investigations [5,11,12,13,19,20,25,26,28] (nine studies, 45%), followed by LN [14,16,22,24] (four studies, 20%), clinical epidemiologic studies [17,27] (two studies, 10%), and other topics, including telemedicine, coronavirus disease 2019 (COVID-19) clinical features, disease classification, and macrophage activation syndrome (MAS) [10,15,18,21,23] (five studies, 25%).

Among treatment studies, multiple reports highlighted the clinical utility of MMF and tacrolimus in maintaining remission and reducing glucocorticoid (GC) exposure. Although current Japanese clinical practice guidance for cSLE does not include biologics, a recent single-center study demonstrated that early combination therapy with belimumab enabled more rapid and smooth tapering of GCs without increasing adverse events [13]. Analyses of national registry and database data further suggested that Japanese children with SLE tend to receive immunosuppressants more frequently and GCs at lower cumulative doses than adults, reflecting a treatment strategy that prioritizes long-term safety and GC-sparing approaches.

### 3.2. Case Reports

Thirty case reports were identified as relevant, and data from 32 cases were extracted (Table 2) [29,30,31,32,33,34,35,36,37,38,39,40,41,42,43,44,45,46,47,48,49,50,51,52,53,54,55,56,57,58]. The average age was 11.9 years (range, 5–18 years), and the ratio of girls to boys was 23:9. LN was present in 20 of the 32 cases [30,31,32,33,35,36,37,38,39,41,43,44,47,48,49,51,55,57,58]. The ISN/RPS classification for the histological classification of LN was established in 2003 and updated in 2018 [8,9]. Many case reports diagnosed LN based on this classification. Most employed the 2003 classification criteria, but none of the case reports published after 2018 explicitly stated that the 2018 classification criteria were applied. The major complications were categorized into seven clinical domains: hematological, neurological, endocrine/metabolic, dermatologic, genetic, infectious, and other systemic manifestations, each exhibiting distinct features and frequencies. Hematological complications were the most frequently reported, occurring in eight cases [29,39,44,47,51,53,55]. These included three cases of thrombotic microangiopathy (TMA), two of which presented as thrombotic thrombocytopenic purpura [39,47,53]. Three children with SLE had antiphospholipid syndrome (APS)-associated complications, including subcapsular renal hematoma after renal biopsy, lower limb arterial occlusion, and combined pulmonary artery thrombosis with deep vein thrombosis [29,51]. Additional hematological manifestations included one case of adrenal hemorrhage associated with lupus anticoagulant hypoprothrombinemia syndrome and one case of mixed autoimmune hemolytic anemia. Neurological involvement was reported in four cases, including two with neuropsychiatric SLE, one with acute necrotizing encephalopathy, and one with vestibular neuritis [35,36,42,46,49]. Four cases presented with endocrine or metabolic abnormalities, including hypophosphatemia, autoimmune thyroiditis, and autoimmune hyperlipidemia [32,33,37,50]. Dermatological manifestations were reported in two cases, both of which required escalation to targeted biologics with anifrolumab due to refractory skin disease [30]. Three cases were associated with genetic conditions, including leukocyte adhesion deficiency type I, C1q deficiency, and prolidase deficiency [45,48,52]. A case of LN caused by prolidase deficiency was treated with rituximab. Infectious complications were reported in two children with SLE: one with cytomegalovirus infection and another with septic arthritis of the pubic symphysis [40,57]. Other manifestations beyond these categories included lupus enteritis and cystitis [43], pancreatitis with walled-off necrosis [54], myositis of the thigh [38], juvenile dermatomyositis [46], and pneumatosis intestinalis with mediastinal emphysema [34]. In one case, the child with cSLE was initially diagnosed with polyarticular juvenile idiopathic arthritis [41]. Most cases fulfilled the 2019 European League Against Rheumatism/ACR classification criteria (EULAR/ACR) [59], the 2012 Systemic Lupus International Collaborating Clinics classification criteria (SLICC) [60], and the JDG; however, a small number did not meet the EULAR/ACR criteria due to negative antinuclear antibody results or missing antibody data [46,49].

### 3.3. Translational Research

Six translational studies were identified (Table 3), most of which focused on MAS. Several studies reported consistently elevated levels of type I interferon (IFN-α), C-X-C motif chemokine ligand 9 (CXCL9), and soluble tumor necrosis factor receptor II (sTNF-RII) in MAS associated with SLE, suggesting a key role for these molecules in disease pathogenesis. Cytokine profiling revealed distinct immunological patterns across different underlying diseases, with SLE-associated MAS showing particularly pronounced activation of interferon signaling pathways [61,62,63]. Analysis of CD169 expression demonstrated increased levels in children with SLE and a strong correlation with IFN-α concentration, suggesting its potential utility as a diagnostic biomarker [64]. Furthermore, macrophage phenotyping studies indicated that GC therapy induced a shift from a pro-inflammatory (M1) to an anti-inflammatory (M2) phenotype, which may serve as an indicator of therapeutic response [65]. In one study, children carrying germline IKAROS family zinc finger 1 (IKZF1) variants showed co-occurrence of autoimmune conditions, including cSLE, implying a genetic contribution to disease susceptibility [66].

### 3.4. Basic Science Studies

A single basic science study using Melanoma Differentiation-Associated gene 5 gain-of-function mouse models demonstrated that type I interferon signaling plays a central role in the development of SLE-like pathology, providing valuable mechanistic insights into disease pathogenesis [67].

### 3.5. Systematic Reviews

The single systematic review focused on patient-reported outcomes and health-related quality of life (HRQOL), areas not fully captured by traditional disease activity measures [68]. It highlighted previously underrecognized unmet needs, including fatigue, sleep disturbances, psychological stress, transitional care challenges, and economic burden. Importantly, it revealed that fatigue and psychological burden may persist even when disease activity is low, underscoring the need for patient-centered support strategies.

### 3.6. Clinical Practice Guidance

The clinical practice guidance provided recommendations for the assessment of disease activity and organ involvement, acute-phase treatment, maintenance therapy following remission induction, and relapse prevention [2,6]. GCs and immunosuppressants remained the mainstay of therapy, whereas discussion of biologics was limited. The guidelines also addressed the epidemiology of cSLE in Japan, reporting a 10-year survival rate of 98.3%, but the rate of survival without organ damage was considerably lower (66.1%). However, these data were based on children with SLE treated between 1995 and 2006, and contemporary data may reflect different trends.

### 3.7. Transition Care Guidance

The Transition Support Guide reported that approximately 525 children with SLE are currently receiving care in Japan and emphasized the need for structured support during the transition to adult medical services [69]. Over the long term, the incidence of cardiovascular events in children with SLE is higher than that in age-matched healthy individuals, highlighting the importance of ongoing monitoring into adulthood. Additionally, SLE is recognized as a risk factor for human papillomavirus infection, and vaccination decisions should be made in consultation with healthcare providers. The guidance also indicated that ultraviolet light exposure can exacerbate disease activity, emphasizing the need for long-term lifestyle modifications and preventive care strategies.

## 4. Discussion

This review summarizes the progress of research on cSLE in Japan over the past decade. Although the total number of publications remains relatively small compared with those on adult SLE, the available studies have steadily enhanced our understanding of disease manifestations, clinical management, and underlying mechanisms.

Clinical research in Japan has primarily been retrospective, but recent years have witnessed a growing number of multicenter and registry-based studies. These collaborative efforts have enabled more accurate data collection and broader epidemiological analyses. The establishment of the national registry (PRICURE) by the Pediatric Rheumatology Association of Japan has been particularly valuable, allowing more systematic tracking of disease characteristics and treatment trends [17]. Continued expansion and utilization of registry data may help identify regional differences and promote greater uniformity in patient management. LN remains a major research focus in Japanese cSLE. Retrospective studies have reported favorable outcomes with immunosuppressive agents, including MMF and tacrolimus, while more recent reports have described early use of biologics, including belimumab, in selected children with SLE [5,12,13,19,26,28]. These findings suggest a gradual shift toward individualized and GC-sparing approaches. Although most studies are still limited in scale, they collectively reflect a growing interest in optimizing long-term safety and minimizing treatment-related complications in children. Simultaneously, the number of domestic multicenter collaborative studies remains limited, and only three international collaborative studies focusing on LN were identified and excluded in this review [70,71,72]. Strengthening the research infrastructure through expanded domestic and international collaboration, therefore, remains an important future challenge.

Case reports have described the diverse clinical spectrum of cSLE, including hematologic, vascular, neurological, endocrine, dermatologic, and genetic complications. Among these, TMA and antiphospholipid antibody-related events have consistently been reported as severe conditions that require timely recognition and appropriate treatment [29,39,47,51,53]. Reports highlighting the effectiveness of anifrolumab and rituximab in children with SLE are also noteworthy [30,46,48]. Several diagnostic frameworks, including the EULAR/ACR, SLICC, and JDG, are currently applied in clinical practice. These frameworks have improved diagnostic consistency; however, most were developed primarily for adult populations. The need for diagnostic and classification criteria that more accurately reflect pediatric disease patterns remains an important consideration for future refinement.

Translational research and basic science studies have provided further insight into the immunopathology of cSLE. Several studies have examined cytokine profiles and interferon pathways, particularly in association with MAS [61,62,63]. Elevated levels of IFN-α, CXCL9, and sTNF-RII have been identified as potential biomarkers for disease activity and treatment response. These findings, though preliminary, illustrate how immunological analyses may contribute to more personalized disease monitoring in the future.

Based on reports from Japan, the clinical characteristics of cSLE are largely consistent with international observations: childhood-onset disease is associated with a higher frequency of renal and multisystem involvement and poorer long-term outcomes compared with adult-onset SLE [4,73].

One notable difference between Japan and Western countries is that LN is more prevalent in Asia and tends to present with greater severity. According to Goto et al., although LN accounts for a relatively small proportion of all pediatric renal biopsy cases (4.5%) [14], representing one of the most clinically important glomerular diseases. Appropriate therapeutic intervention has been shown to improve disease activity markers such as anti–double-stranded DNA antibody titers and hypocomplementemia, as reported by Hara et al. [26]. Furthermore, Ikezumi et al. demonstrated that GC therapy is associated with a phenotypic shift of intrarenal macrophages from a pro-inflammatory (M1) to an anti-inflammatory (M2) profile [65], providing immunological support for the validity of current treatment strategies for SLE.

Despite these advances, gaps between clinical practice and treatment strategies in Japan and those recommended internationally appear to persist. The 2023 update of the EULAR recommendations for the management of SLE advocates initiation of HCQ at diagnosis in the absence of contraindications, the use of immunosuppressive and biologics as appropriate, and the administration of GCs at the lowest effective dose for the shortest possible duration [74]. In the United States, prescription rates among newly diagnosed children with SLE have been reported to be comparable, at 64.4% for HCQ and 62.0% for GCs [75]. Conversely, a nationwide database analysis by Matsushita et al. revealed that, among Japanese children aged 0–14 years with SLE, the prescription rate of HCQ was notably low at 22.7%, whereas GCs were prescribed in 78.8% of children [5]. The use of biologics was limited in this study (rituximab 5%, belimumab 4.2%) [5]. In Japan, HCQ was approved in 2015 and MMF in 2016 as treatments for SLE and LN, including childhood-onset cases, followed by belimumab in 2019 and rituximab in 2023. Anifrolumab remains unapproved for pediatric use in Japan. The low utilization of HCQ may be partly attributable to historical concerns regarding chloroquine-associated retinopathy, which led to prolonged market withdrawal of chloroquine and the establishment of GC-centered treatment strategies in clinical practice during that period. Regarding long-term outcomes, a nationwide survey conducted in 2009 reported a 10-year cumulative survival rate of 98.3% among patients with cSLE, demonstrating a marked improvement compared with earlier surveys (92.3%). However, the 10-year survival rate without permanent organ damage or sequelae remained limited to 66.1%, indicating that many patients continue to live with chronic organ damage or treatment-related complications [2]. Although Kishi et al. reported a reduction in daily GC doses and a gradual increase in the use of combination therapy with immunosuppressive agents between 2009 and 2018, a more recent report by Matsushita et al. in 2025 showed high prescription rates of antihypertensive and anti-osteoporotic medications among children aged 0–14 years with SLE [5,20]. These findings indicate that concerns regarding GC exposure continue to be discussed in the context of cSLE care in Japan. Accordingly, minimizing GC exposure has increasingly been recognized as an important therapeutic consideration in recent studies. Discussion of this objective has focused primarily on raising clinician awareness of potential GC-related adverse effects, rather than on direct evidence of observed adverse events, together with the use of treatment strategies that combine multiple immunosuppressive agents, including HCQ. As the use of biologics in Japanese cSLE remains limited, their potential contribution to strategies aimed at reducing GC exposure remains an important area for future investigation.

The healthcare context for cSLE in Japan mirrors challenges reported internationally: while care provided by pediatric nephrologists is essential, care provided by pediatric rheumatologists is equally critical, and the limited number of pediatric rheumatologists restricts equitable access to specialized care and research opportunities for children with SLE. In contrast, recent studies from Europe and other regions have increasingly focused on treat-to-target strategies and the assessment of HRQOL in cSLE [76], whereas these themes remain underrepresented in Japan. From the perspective of linking disease activity control with patient-centered outcomes, areas such as HRQOL, long-term organ outcomes, and transition care from pediatric to adult services represent important priorities for future research in Japan. Given that cSLE often develops during adolescence, further investigation into psychosocial factors, educational impact, and patient-reported outcomes would enable a more comprehensive understanding of disease burden.

Furthermore, the relatively small number and geographic maldistribution of pediatric rheumatologists in Japan pose practical limitations on conducting large-scale studies and establishing uniform nationwide care. Nevertheless, revision of domestic guidance incorporating recent Japanese research findings may strengthen collaboration between specialists and general pediatricians, facilitate earlier diagnosis and appropriate referral, and promote continuity of care. Such efforts are also expected to support greater participation in multicenter collaborative research and contribute to more standardized management of cSLE across Japan.

This review has some limitations. First, it included only English-language articles indexed in PubMed. As the literature search was limited to a single database, relevant studies indexed in other databases may not have been identified. In addition, Japanese-language publications, which may contain clinically relevant information specific to childhood-onset SLE in Japan, were not included. Second, this review focused on studies in which Japanese pediatric cohorts were explicitly reported. Consequently, multinational or international collaborative studies without extractable Japan-specific pediatric data were excluded, including three international studies focusing on lupus nephritis [70,71,72]. This approach may have introduced selection bias and may limit the representativeness and generalizability of the findings. Accordingly, the conclusions of this review pertain strictly to studies with explicitly reported Japan-specific cohorts and should be interpreted with caution. Third, most clinical studies included in this review were retrospective, single-center, and observational in design, often with small sample sizes. Importantly, no formal quality assessment or risk-of-bias scoring framework (such as the Newcastle–Ottawa Scale) was applied, as this work was conceived as a narrative review rather than a systematic evidence synthesis. The absence of standardized quality evaluation limits the ability to compare studies or to draw graded conclusions regarding treatment efficacy or clinical effectiveness. Thus, interpretations related to treatment strategies, including GC-sparing approaches, should be regarded as descriptive and hypothesis-generating rather than confirmatory. Observed differences in treatment practices or reported outcomes may reflect underlying study design limitations, institutional practices, or patient heterogeneity rather than true comparative effectiveness.

Future prospective, multicenter studies with standardized outcome measures and formal quality assessment are needed to strengthen the pediatric-specific evidence base.

## 5. Future Research Directions and Unmet Needs

From the perspective of linking disease activity control with patient-centered outcomes, HRQOL, long-term organ outcomes, and transition care from pediatric to adult services represent important priorities for future research in Japan. In the management of cSLE in Japan, GC-related adverse effects remain an important area of concern, underscoring the need to further refine and implement GC-sparing approaches. Notably, although the current utilization of HCQ and biologics remains relatively low in Japan, the appropriate combined use of these therapies should be further evaluated in carefully selected children with SLE.

In addition, large-scale multicenter studies focusing specifically on cSLE remain limited in Japan, and strengthening the research infrastructure through expanded domestic and international collaboration continues to be an important challenge. Notably, in this review, no genetic epidemiology studies or genome-wide association studies specifically targeting Japanese children with SLE were identified. Given the increasing application of genetic approaches in SLE research worldwide [77], this represents an important unmet need and a promising direction for future research in Japan.

## 6. Conclusions

Research on cSLE in Japan has substantially advanced, leading to a deeper understanding of its clinical manifestations, management strategies, and underlying mechanisms. Nevertheless, treatment-related complications and long-term organ damage remain major challenges. To address these issues, future efforts may focus on clarifying GC-sparing approaches in a manner that is easily understood by general clinicians and allows for consistent implementation across clinical settings in Japan. Currently, domestic multicenter studies and international collaborative research remain limited, underscoring the need for further expansion of such efforts. The accumulation of such studies may help generate evidence that more accurately reflects real-world clinical practice and the characteristics of Japanese children with SLE, thereby providing background information to support future discussions on clinical practice guidelines in Japan.

## Figures and Tables

**Table 1:** Summary of published cSLE clinical studies in Japan (2015–2025).

No.	Author (Year)	Study Type	Participating Institution	Sample Size	Purpose	Key Findings
1	Inoue et al. (2025) [10]	Retrospective observational study	Multicenter	Pediatric rheumatologists (*n* = 128)	To evaluate the perceived necessity, advantages, and challenges of telemedicine in cSLE management.	Among the surveyed physicians 76.6% reported patients traveling over an hour for visits. Telemedicine benefits were noted by most respondents, including reduced travel and consultation time (92.2%), with 80.2% supporting its use, particularly in remission or stable disease (76.3%).
2	Matsushita et al. (2025) [5]	Retrospective observational study	National database	cSLE (*n* = 782)	To characterize age-specific treatment patterns of SLE in Japan.	Pediatric patients showed lower GC use (78.8%) than did adults (89.5% in ages 40–64 years) but higher MMF use (49.2% vs. 12.2%). Use of biologics (rituximab or belimumab) was higher in children (4–5%) than in adults (<1%).
3	Saito et al. (2025) [11]	Retrospective observational study	Multicenter	Pediatric diseases (*n* = 249; LN, *n* = 17)	To identify factors affecting MMF pharmacokinetics in pediatric patients.	MMF clearance was significantly influenced by body weight, serum albumin, kidney transplant status, and concomitant PPI use.
4	Tanaka et al. (2024) [12]	Retrospective observational study	Single center	LN (*n* = 13)	To evaluate long-term outcomes of tacrolimus-based therapy.	Over a 10-year follow-up period, SLEDAI scores, complement levels, anti-dsDNA antibody levels, and proteinuria improved within 1 year and remained stable thereafter. Over 10 years, the median prednisolone (PSL) dose decreased from 18.7 mg/day at treatment initiation to 3.5 mg/day.
5	Hashimoto et al. (2024) [13]	Retrospective observational study	Single center	cSLE (*n* = 6)	To investigate the efficacy of early belimumab combination therapy.	Patients receiving GCs in combination with MMF and HCQ were divided into two groups: those who initiated intravenous belimumab within 1 month and those who did not receive belimumab. Although disease activity improved in both groups, patients who received early belimumab initiation achieved more rapid and smoother tapering of GCs.
6	Goto et al. (2023) [14]	Retrospective observational study	Multicenter (Registry-based)	Pediatric renal diseases (*n* = 3526; LN, *n* = 160)	To describe renal biopsy diagnoses in Japan.	IgA nephropathy (36.1%) and minimal change disease (17.6%) were most frequent in pediatric biopsies, while LN accounted for 4.5%.
7	Wakiguchi et al. (2023) [15]	Retrospective observational study	Multicenter	Pediatric rheumatic diseases (*n* = 156; cSLE, *n* = 37)	To examine COVID-19 severity in pediatric rheumatic disease.	Most infections were asymptomatic or mild (97%), with no severe or fatal outcomes. Use of immunosuppressants or biologics did not correlate with increased infection severity.
8	Nishimura et al. (2023) [16]	Retrospective observational study	Single center	LN (*n* = 14), Dent disease (*n* = 23)	To compare urinary β2-MG/TP ratios across pediatric kidney diseases.	The β2-MG/TP ratio was significantly higher in Dent disease than in LN, distinguishing tubulointerstitial from glomerular pathologies and highlighting its diagnostic utility.
9	Narazaki et al. (2023) [17]	Retrospective observational study	Multicenter (Registry-based)	Pediatric rheumatic diseases (*n* = 402; cSLE, *n* = 40)	To characterize demographic and diagnostic features of pediatric SLE.	Median onset age was 12.1 years, with a girl: boy ratio of 3.4:1. Diagnosis was established earlier in SLE cases than in juvenile dermatomyositis and Sjögren’s disease cases. Kidney biopsy was performed in 81.8% of SLE cases.
10	Ohara et al. (2022) [18]	Retrospective observational study	Single center	cSLE (*n* = 53), disease controls (*n* = 53)	To assess the performance of EULAR/ACR criteria in children.	The 2019 criteria demonstrated high sensitivity and specificity, including early disease phases (≤3 months). Performance surpassed ACR and SLICC criteria in diagnostic balance.
11	Nishi et al. (2022) [19]	Retrospective observational study	Single center	cSLE (*n* = 31)	To evaluate GC tapering and discontinuation.	GC withdrawal was achieved in 61% of patients, with cumulative discontinuation rates of 26% at 3 years, 63% at 5 years, and 95% at 10 years. Early MMF initiation and prompt transition to maintenance were associated with sustained remission.
12	Kishi et al. (2022) [20]	Retrospective observational study	National database	cSLE (*n* = 182)	To examine treatment trends over a decade.	Oral PSL was administered to >97% of patients, with the maximum dose declining over time. Intravenous cyclophosphamide use declined post-2016, while MMF and HCQ use increased. Combination therapy became more common.
13	Sato et al. (2019) [21]	Retrospective observational study	Single center	cSLE (*n* = 46; MAS, *n* = 11)	To describe MAS as an initial manifestation.	MAS occurred at onset in 23.9% of cases. Early GCs with immunosuppressants were commonly used, and laboratory features were described.
14	Ishimori et al. (2019) [22]	Retrospective observational study	Multicenter	LN (*n* = 36)	To characterize LN presenting with acute kidney injury at onset.	Nineteen percent of patients presented with acute kidney injury displaying higher disease activity and proliferative pathology. Six of seven recovered renal function within 6 months, while one progressed to end-stage kidney disease.
15	Kawano et al. (2017) [23]	Retrospective observational study	Single center	cSLE (*n* = 29)	To identify thyroid cancer risk factors in cSLE.	Three patients with cSLE developed thyroid cancer; lymphadenopathy/splenomegaly, weight loss, urinary granular casts, and anemia were identified as predisposing factors.
16	Wakiguchi et al. (2017) [24]	Retrospective observational study	Single center	cSLE (*n* = 45)	To examine the association of complement levels with LN.	In silent LN, 24% had class III lesions. C3 levels correlated with pathology class but not in overt LN. C4 showed no significant difference.
17	Fujinaga and Nishino (2017) [25]	Retrospective observational study	Single center	LN (*n* = 6)	To evaluate the impact of therapeutic drug monitoring on long-term MMF therapy.	Median treatment duration was 9.8 years. The SLEDAI score decreased from 7 to 0, with no renal flares or chronic kidney disease observed. PSL dose decreased from 10.8 to 1.8 mg/day. Maintaining MMF trough levels at 2–5 μg/mL was associated with disease stability.
18	Hara et al. (2015) [26]	Retrospective observational study	Multicenter	cSLE (*n* = 115)	To characterize the nationwide use, efficacy, and safety of MMF.	GC dose decreased significantly, complement levels increased, and anti-dsDNA titers decreased. Adverse events occurred in 24 cases (4.3/100 person-years).
19	Kawasaki et al. (2015) [27]	Retrospective observational study	Multicenter	cSLE (*n* = 37)	To examine incidence, clinical features, treatment, and outcomes over 35 years.	While incidence remained stable, treatment was initiated earlier, immunosuppressant use increased, remission rates rose to 94%, and renal failure was eliminated.
20	Kizawa et al. (2015) [28]	Retrospective observational study	Single center	LN (*n* = 9)	To assess 12-month maintenance therapy with MMF plus GCs following induction therapy.	Eighty-nine percent of patients showed histologic improvement, with the SLEDAI score decreasing from 16.2 to 2.0, complement and anti-dsDNA improving, and no relapses. GC doses were significantly reduced while maintaining remission.

ACR, The 1997 American College of Rheumatology classification criteria; β2-MG/TP, β2-Microglobulin/total protein; COVID-19, Coronavirus disease 2019; cSLE, Childhood-onset systemic lupus erythematosus; EULAR/ACR, The 2019 European League Against Rheumatism and American College of Rheumatology classification criteria; GC, Glucocorticoid; HCQ, Hydroxychloroquine; IgA, Immunoglobulin A; LN, Lupus nephritis; MAS, Macrophage activation syndrome; MMF, Mycophenolate mofetil; PSL, Prednisolone; SLE, Systemic lupus erythematosus; SLEDAI, Systemic Lupus Erythematosus Disease Activity Index; SLICC, The 2012 Systemic Lupus International Collaborating Clinics classification criteria.

**Table 2:** Summary of published cSLE case reports in Japan (2015–2025).

No.	Author (Year)	Age/ Sex	Criteria/ Guidance Met ^(1)^	Major Complications	Key Findings
1	Uechi et al. (2025) [29]	12 y/F	EULAR/ACR SLICC JDG	APS, popliteal artery occlusion	The patient presented with intermittent claudication and toe discoloration and was diagnosed with SLE and APS complicated by popliteal artery occlusion. Early vascular assessment and combination therapy with GCs, MMF, HCQ, and anticoagulation were administered to preserve limb function.
2	Shmizu et al. (2025) [30]	12 y/M	EULAR/ACR SLICC JDG	ILD, refractory skin lesions, MAS	The patient developed MAS and was treated with dexamethasone palmitate, cyclosporine, and ruxolitinib. Long-term disease control was achieved with ruxolitinib and PSL. The patient’s skin manifestations were unresponsive to ruxolitinib but showed marked improvement after initiating anifrolumab.
3	Shmizu et al. (2025) [30]	12 y/F	EULAR/ACR SLICC JDG	Refractory skin lesions, LN class II	The patient received methylPSL pulse therapy followed by PSL and mizoribine. However, skin lesions remained GC-dependent despite additional treatment with tacrolimus, MMF, HCQ, and belimumab. Initiation of anifrolumab led to dramatic improvement.
4	Kise and Uehara (2025) [31]	18 y/F	NA	LN class IV	The patient received PSL (6 mg), MMF (500 mg), and belimumab. Three months later, belimumab was switched to intravenous administration to reduce injection pain. Following PSL discontinuation, she remained relapse-free for 31 months.
5	Kise and Uehara (2025) [31]	15 y/M	NA	LN class IV	After 3 years of remission, the patient’s SLE worsened due to poor adherence. She received PSL, MMF, and IV belimumab. Following PSL tapering, a flare occurred, which responded to methylPSL pulse therapy. PSL was gradually discontinued, and she remained relapse-free for 8 months before transfer to adult care.
6	Wakatsuki et al. (2023) [32]	14 y/F	EULAR/ACR SLICC JDG	Atrophic autoimmune thyroiditis, LN class II	The patient with severe hypothyroidism and class II LN presented with anorexia, numbness, and edema. Myxedema-related effusions resolved with hormone replacement and immunosuppressive therapy, resulting in remission.
7	Kawaguchi and Inamo (2023) [33]	11 y/F	EULAR/ACR SLICC JDG	Hypophosphatemia, LN class II	The patient exhibited marked hypophosphatemia and elevated fibroblast growth factor 23 levels at onset. Treatment with methylPSL pulse therapy, oral PSL, MMF, HCQ, and phosphate supplementation rapidly normalized phosphate levels without recurrence.
8	Uda et al. (2023) [34]	13 y/M	NA	Pneumatosis cystoides intestinalis, pneumomediastinum	During SLE treatment, the patient developed mild neck pain, abdominal distension, and subcutaneous emphysema. Imaging revealed mediastinal emphysema and pneumatosis intestinalis without perforation. Conservative management with oxygen therapy, antibiotics, bowel rest, total parenteral nutrition, and GC tapering resulted in full resolution.
9	Kaneko et al. (2022) [35,36]	14 y/F	EULAR/ACR SLICC JDG	Vestibular neuritis, LN class III(C) + V	The patient presented with prolonged dizziness and hearing loss. MethylPSL pulse therapy was followed by oral PSL and azathioprine, leading to the resolution of systemic manifestations and vestibular symptoms.
10	Shimazaki et al. (2022) [37]	12 y/F	EULAR/ACR SLICC JDG	Hypophosphatemia, autoimmune thyroiditis, LN class V	The patient presented with hypophosphatemia and elevated fibroblast growth factor 23 levels at diagnosis. Treatment with MMF and GCs improved complement levels and reduced fibroblast growth factor 23 levels.
11	Hoshi et al. (2022) [38]	14 y/F	EULAR/ACR SLICC JDG	Myositis, LN class II	The patient’s MRI revealed high signal intensity in the quadriceps and sartorius muscles. The patient was treated with methylPSL pulse therapy, PSL, HCQ, and MMF, resulting in clinical and radiological improvement, with normalization of serum interferon-alpha levels.
12	Endo et al. (2022) [39]	7 y/M	EULAR/ACR SLICC JDG	TTP, LN class III	The patient presented with fever, purpura, and nausea. Laboratory findings confirmed SLE-associated TTP. Plasma exchange and methylPSL pulse therapy improved platelet counts and renal function. Maintenance therapy with cyclophosphamide and HCQ sustained remission.
13	Shimizu et al. (2022) [40]	14 y/F	NA	Septic arthritis of the pubic symphysis	The patient developed septic arthritis of the pubic symphysis, presenting with sudden-onset pubic pain and restricted hip motion during remission. Treatment with vancomycin, intravenous immunoglobulin, and a 4-week course of oral clindamycin resolved the infection without recurrence.
14	Kama et al. (2022) [41]	12 y/F	EULAR/ACR SLICC JDG	Polyarthritis, LN class II	The patient initially presented with chronic polyarthritis and elevated anti-cyclic citrullinated peptide antibody levels, leading to a provisional diagnosis of polyarticular juvenile idiopathic arthritis. Six months later, SLE was diagnosed. Remission was achieved after two courses of methylPSL pulse therapy, followed by PSL and HCQ.
15	Korenaga et al. (2021) [42]	15 y/F	EULAR/ACR SLICC JDG	NP-SLE, Acute necrotizing encephalopathy-like encephalopathy	The patient presented with seizures, altered consciousness, and fever. MRI revealed bilateral thalamic high-signal lesions resembling acute necrotizing encephalopathy. Treatment with methylPSL pulse therapy and cyclophosphamide improved consciousness. One year later, only mild cognitive impairment remained.
16	Shimizu et al. (2021) [43]	11 y/F	EULAR/ACR SLICC JDG	Lupus enteritis, lupus cystitis, LN class II	The patient presented with persistent abdominal pain, watery diarrhea, and weight loss. Imaging revealed bowel wall thickening and mesenteric vascular proliferation. PSL 1 mg/kg/day rapidly improved symptoms within 2 weeks.
17	Sakamoto et al. (2021) [44]	9 y/M	EULAR/ACR SLICC JDG	LAHPS, adrenal hemorrhage, LN class unknown	The patient initially presented with abdominal pain and coagulopathy, leading to a diagnosis of lupus anticoagulant–hypoprothrombinemia syndrome complicated by bilateral adrenal hemorrhage. One year later, SLE developed, which responded well to immunosuppressive therapy, resulting in remission.
18	Matsumura et al. (2021) [45]	12 y/F	EULAR/ACR SLICC JDG	C1q deficiency	The patient had refractory SLE due to C1q deficiency. Allogeneic bone marrow transplantation achieved complete donor chimerism and resolution of clinical symptoms, with normalization of C1q levels.
19	Uejima et al. (2021) [46]	9 y/F	SLICC JDG	Juvenile dermatomyositis, ILD, NP-SLE	The patient was diagnosed with overlap syndrome of juvenile dermatomyositis and SLE complicated by rapidly progressive ILD. Later, NP-SLE developed. Intensive immunosuppressive therapy, including rituximab and plasma exchange, led to clinical improvement.
20	Kaneda et al. (2020) [47]	5 y/F	EULAR/ACR SLICC JDG	TMA, LN class IV-S(A)	The patient presented with fever, edema, cytopenia, hemolytic anemia, thrombocytopenia, and renal dysfunction. Intensive immunosuppressive therapy, including methylPSL, plasma exchange, MMF, and cyclophosphamide, achieved disease control.
21	Sato et al. (2020) [48]	15 y/M	EULAR/ACR SLICC JDG	Prolidase deficiency, LN class IV	The patient presented with malar rash, hematuria, positive anti-dsDNA antibodies, and hypocomplementemia. High-dose GCs and four doses of rituximab led to improvement in proteinuria, complement levels, and skin ulcers, with no relapse observed.
22	Doi et al. (2019) [49]	17 y/F	SLICC JDG	NP-SLE, LN class IV-G(A)	SLE was diagnosed at age 14. During maintenance therapy, relapse occurred, followed by severe MMF toxicity manifesting as seizures, acute kidney injury, leukopenia, and thrombocytopenia. After MMF was discontinued, renal function and hematological parameters normalized, and no neurological sequelae were observed.
23	Inoue et al. (2019) [50]	11 y/F	EULAR/ACR SLICC JDG	Hyperlipidemia, anti-apolipoprotein C-II autoantibody	The patient presented with malar rash and polyarthritis. Laboratory findings revealed severe hypertriglyceridemia, decreased high-density lipoprotein cholesterol and low-density lipoprotein cholesterol levels, and the presence of anti-apolipoprotein C-II autoantibodies. Treatment with bezafibrate, PSL, and MMF normalized triglyceride levels within 2 weeks.
24	Nagata et al. (2018) [51]	8 y/M	EULAR/ACR SLICC JDG	APS, renal hematoma, LN class III(A),	The patient was initially diagnosed with APS and developed SLE 14 months later. Renal biopsy confirmed class III LN, and a large subcapsular renal hematoma occurred 9 days after the procedure.
25	Nagata et al. (2018) [51]	13 y/M	EULAR/ACR SLICC JDG	APS, deep vein thrombosis, pulmonary artery thrombosis, LN class III(A)	The patient was diagnosed with SLE and APS. Renal biopsy revealed LN and TTP. Six days after biopsy, deep vein thrombosis and pulmonary embolism occurred.
26	Kurosawa et al. (2018) [52]	7 y/F	EULAR/ACR SLICC JDG	Leukocyte adhesion deficiency type 1, necrotizing ulcer	The patient developed necrotizing ulcers following Bacillus Calmette–Guérin vaccination and was diagnosed with leukocyte adhesion deficiency type 1. One year later, SLE was diagnosed. Hematopoietic stem cell transplantation from a human leukocyte antigen-matched sibling achieved long-term remission without further immunosuppressive therapy.
27	Shingu et al. (2017) [53]	8 y/M	EULAR/ACR SLICC JDG	TTP	The patient presented with fever, thrombocytopenia, Coombs-negative hemolytic anemia, and neurological symptoms. Severe ADAMTS13 deficiency and anti-ADAMTS13 antibodies were detected. Despite repeated relapses, combination therapy with plasma exchange, GCs, cyclophosphamide, and azathioprine achieved sustained remission.
28	Sato et al. (2016) [54]	13 y/F	EULAR/ACR SLICC JDG	MAS, acute pancreatitis, walled-off pancreatic necrosis	The patient was diagnosed with SLE and MAS. Acute pancreatitis developed 2 days after initiating high-dose GCs. Walled-off necrosis and infected pancreatic abscess required multiple endoscopic necrosectomies and stent placement. Long-term disease control was achieved with PSL and MMF.
29	Hirano et al. (2016) [55]	9 y/F	EULAR/ACR SLICC JDG	Mixed-type autoimmune hemolytic anemia, pericarditis, LN class II	The patient presented with autoimmune hemolytic anemia, skin rash, oral ulcers, serositis, and renal involvement. Treatment with GCs and MMF led to GC-free remission, which was maintained for 18 months.
30	Nakagishi et al. (2016) [56]	15 y/F	EULAR/ACR SLICC JDG	MAS	Four days after starting PSL, the patient with cSLE developed MAS with fever, rash, pancytopenia, hyperferritinemia, and liver injury. She was unresponsive to methylPSL and cyclosporine but improved rapidly after dexamethasone and remains in remission.
31	Yamazaki et al. (2015) [57]	12 y/F	EULAR/ACR SLICC JDG	Cytomegalovirus infection, LN class III	The patient presented with lymphadenopathy and liver dysfunction, later developing fever and malar rash. Concurrent primary cytomegalovirus infection was confirmed. Combined therapy with GCs, cyclophosphamide, and ganciclovir led to complete remission without relapse.
32	Kise et al. (2015) [58]	13 y/F	EULAR/ACR SLICC JDG	LN class IV-G(A) + V	The patient was diagnosed with SLE and LN at age 11 and experienced a relapse due to poor adherence. Combination therapy with MMF and tacrolimus successfully induced remission and improved renal function.

^(1)^ Among the criteria/guidance listed in the table, items underlined were explicitly documented in the original articles as diagnostic evidence, whereas those without underlining were inferred to fulfill the criteria/guidance based on the clinical manifestations and laboratory findings described in the case reports. ADAMTS13, ADAM metallopeptidase with thrombospondin type 1 motif 13; APS, Antiphospholipid syndrome; cSLE, Childhood-onset systemic lupus erythematosus; EULAR/ACR, The 2019 European League Against Rheumatism and American College of Rheumatology classification criteria; GC, Glucocorticoid; HCQ, Hydroxychloroquine; ILD, Interstitial lung disease; JDG, The Japanese diagnostic guidance for childhood-onset systemic lupus erythematosus; LAHPS, Lupus anticoagulant–hypoprothrombinemia syndrome; LN, Lupus nephritis; MAS, Macrophage activation syndrome; MMF, Mycophenolate mofetil; MRI, Magnetic resonance imaging; NA, Not available; NP-SLE, Neuropsychiatric systemic lupus erythematosus; PSL, Prednisolone; SLE, Systemic lupus erythematosus; SLICC, The 2012 Systemic Lupus International Collaborating Clinics classification criteria; TMA, Thrombotic microangiopathy; TTP, Thrombotic thrombocytopenic purpura.

**Table 3:** Summary of published cSLE translational studies in Japan (2015–2025).

No.	Author (Year)	Study Type	Participating Institution	Sample Size	Purpose	Key Findings
1.	Kaneko et al. (2025) [61]	Retrospective observational study	Multicenter	Cytokine storm syndrome (*n* = 143; SLE with MAS, *n* = 10), healthy controls (*n* = 22)	To characterize cytokine profiles in cytokine storm syndrome and identify disease-specific patterns.	This study measured 48 serum cytokines in patients with cytokine storm syndrome. Five disease-related clusters were identified, with IFN-α levels markedly elevated in SLE-associated MAS, implicating the type I interferon pathway in disease pathogenesis.
2.	Sakumura et al. (2023) [64]	Retrospective observational study	Single center	Pediatric inflammatory diseases (*n* = 43; cSLE, *n* = 10)	To assess the clinical significance of CD169 expression.	This study investigated the clinical significance of CD169 expression on monocytes in pediatric inflammatory diseases, including 10 patients with SLE. Surface CD169 on CD14^+^ monocytes and soluble CD169 levels were quantified and correlated with type I interferon activity. The SLE group showed significantly higher CD169 expression levels than those of other febrile disease groups. Notably, IFN-α concentrations and soluble CD169 levels exhibited strong positive correlations.
3.	Ikezumi et al. (2021) [65]	Retrospective observational study	Single center	LN (*n* = 32)	To examine GC effects on macrophage polarization.	This study examined the effect of GC therapy on macrophage polarization in pediatric LN. Seventeen GC-naïve patients were compared with fifteen who had received GCs prior to biopsy. In untreated patients, M1 macrophages predominated, whereas GC-treated patients exhibited a shift toward M2 macrophage dominance, reflected by an approximately six-fold increase in the M2/M1 ratio.
4.	Mizuta et al. (2021) [62]	Retrospective observational study	Multicenter	Pediatric rheumatic diseases (*n* = 278; cSLE, *n* = 12; cSLE with MAS, *n* = 5)	To compare cytokine profiles across MAS etiologies.	This study compared serum cytokine profiles among patients with MAS secondary to various rheumatic diseases, including five pediatric patients with SLE-associated MAS. Cytokine measurements and clustering analyses revealed that SLE-associated MAS was characterized by higher IFN-γ, IL-18, CXCL9, and sTNF-RII levels than those of MAS associated with other diseases, indicating a disease-specific inflammatory pattern.
5.	Usami et al. (2019) [63]	Retrospective observational study	Single center	cSLE with MAS (*n* = 4), cSLE without MAS (*n* = 4)	To analyze cytokine changes in SLE-associated MAS.	This study simultaneously measured 174 serum cytokines in eight pediatric patients with SLE, including four with MAS, to analyze MAS-associated cytokine changes. Thirty-one cytokines were significantly elevated during MAS compared to active SLE without MAS, with particularly marked increases in CXCL9 and sTNF-RII levels, reflecting heightened disease activity in SLE-associated MAS.
6.	Hoshino et al. (2017) [66]	Retrospective observational study	Multicenter	IKZF1 mutation (heterozygous) (*n* = 9)	To describe immune abnormalities in IKZF1-mutated patients.	This study analyzed hematopoietic and immunological features in nine pediatric patients with heterozygous germline IKZF1 mutations. Four patients developed autoimmune diseases, including one with systemic lupus erythematosus. These findings suggest that germline IKZF1 mutations may predispose individuals to immune dysregulation and the development of autoimmune diseases, such as SLE.

cSLE, Childhood-onset systemic lupus erythematosus; CXCL9, C-X-C motif chemokine ligand 9; GC, Glucocorticoid; IFN, Interferon; IKZF1, IKAROS family zinc finger 1; IL, Interleukin; LN, Lupus nephritis; MAS, Macrophage activation syndrome; SLE, Systemic lupus erythematosus; sTNF-RII, Soluble tumor necrosis factor receptor II; TNF, Tumor necrosis factor.

## Data Availability

No new data were generated or analyzed in this study.

## Abbreviations

The following abbreviations are used in this manuscript:

ACR	The 1997 American College of Rheumatology classification criteria
ADAMTS13	ADAM metallopeptidase with thrombospondin type 1 motif 13
APS	Antiphospholipid syndrome
β2-MG/TP	β2-Microglobulin/total protein
COVID-19	Coronavirus disease 2019
cSLE	Childhood-onset systemic lupus erythematosus
CXCL9	C-X-C motif chemokine ligand 9
EULAR/ACR	The 2019 European League Against Rheumatism and American College of Rheumatology classification criteria
GC	Glucocorticoid
HCQ	Hydroxychloroquine
HRQOL	Health-related quality of life
IFN	Interferon
IgA	Immunoglobulin A
IKZF1	IKAROS family zinc finger 1
IL	Interleukin
ILD	Interstitial lung disease
ISN	International Society of Nephrology
JDG	The Japanese diagnostic guidance for childhood-onset systemic lupus erythematosus
LAHPS	Lupus anticoagulant–hypoprothrombinemia syndrome
LN	Lupus nephritis
MAS	Macrophage activation syndrome
MeSH	Medical Subject Headings
MMF	Mycophenolate mofetil
MRI	Magnetic resonance imaging
NP-SLE	Neuropsychiatric systemic lupus erythematosus
PRICURE	Pediatric Rheumatology International Collaboration Unit Registry
PSL	Prednisolone
RPS	Renal Pathology Society
SLE	Systemic lupus erythematosus
SLEDAI	Systemic lupus erythematosus disease activity index
SLICC	The 2012 Systemic Lupus International Collaborating Clinics classification criteria
sTNF-RII	Soluble tumor necrosis factor receptor II
TMA	Thrombotic microangiopathy
TTP	Thrombotic thrombocytopenic purpura
